# Mitochondrial complex I inhibition triggers NAD^+^-independent glucose oxidation via successive NADPH formation, “futile” fatty acid cycling, and FADH_2_ oxidation

**DOI:** 10.1007/s11357-023-01059-y

**Published:** 2024-01-25

**Authors:** Roman Abrosimov, Marius W. Baeken, Samuel Hauf, Ilka Wittig, Parvana Hajieva, Carmen E. Perrone, Bernd Moosmann

**Affiliations:** 1 Institute for Pathobiochemistry, University Medical Center of the Johannes Gutenberg University, Mainz, Germany; 2 Nucleic Acid Chemistry and Engineering Unit, Okinawa Institute of Science and Technology Graduate University, Onna, Okinawa Japan; 3 Institute for Cardiovascular Physiology, Goethe University, Frankfurt, Germany; 4 Institute for Translational Medicine, MSH Medical School, Hamburg, Germany; 5 Orentreich Foundation for the Advancement of Science, Cold Spring-On-Hudson, NY USA

**Keywords:** Diabetes, FGF21, Metformin, Methionine restriction, NADH dehydrogenase, Peroxisome proliferator-activated receptor

## Abstract

Inhibition of mitochondrial complex I (NADH dehydrogenase) is the primary mechanism of the antidiabetic drug metformin and various unrelated natural toxins. Complex I inhibition can also be induced by antidiabetic PPAR agonists, and it is elicited by methionine restriction, a nutritional intervention causing resistance to diabetes and obesity. Still, a comprehensible explanation to why complex I inhibition exerts antidiabetic properties and engenders metabolic inefficiency is missing. To evaluate this issue, we have systematically reanalyzed published transcriptomic datasets from MPP-treated neurons, metformin-treated hepatocytes, and methionine-restricted rats. We found that pathways leading to NADPH formation were widely induced, together with anabolic fatty acid biosynthesis, the latter appearing highly paradoxical in a state of mitochondrial impairment. However, concomitant induction of catabolic fatty acid oxidation indicated that complex I inhibition created a “futile” cycle of fatty acid synthesis and degradation, which was anatomically distributed between adipose tissue and liver in vivo. Cofactor balance analysis unveiled that such cycling would indeed be energetically futile (-3 ATP per acetyl-CoA), though it would not be redox-futile, as it would convert NADPH into respirable FADH_2_ without any net production of NADH. We conclude that inhibition of NADH dehydrogenase leads to a metabolic shift from glycolysis and the citric acid cycle (both generating NADH) towards the pentose phosphate pathway, whose product NADPH is translated 1:1 into FADH_2_ by fatty acid cycling. The diabetes-resistant phenotype following hepatic and intestinal complex I inhibition is attributed to FGF21- and GDF15-dependent fat hunger signaling, which remodels adipose tissue into a glucose-metabolizing organ.

**Supplementary Information:**

The online version contains supplementary material available at 10.1007/s11357-023-01059-y.

## Introduction

Fatty acid cycling, defined as the parallel synthesis and degradation of fatty acids in the same cell or organism, is a repeatedly described metabolic phenomenon whose objective has remained elusive. Fatty acid cycling produces a negative energy balance, indicating that its purpose will arguably be related to regulation, communication between metabolic pathways or organs, reduction of toxicity, thermogenesis, or a combination of these factors [24, 82, 85]. Notably, fatty acid cycling has primarily been described as an adipose tissue phenomenon associated with antidiabetic interventions, either pharmacological [82], nutritional [85], or thermal [43, 113]. In several cases, these antidiabetic interventions were caused or accompanied by mitochondrial complex I inhibition, suggesting that both phenomena may be causally related.

Metformin is a metabolic modulator that is widely prescribed for type II diabetes treatment [91, 92]. Most of its numerous metabolic effects appear to originate from complex I inhibition in the liver and intestine [17, 21, 39, 91]. Metformin treatment has been linked to increased overall fatty acid oxidation, especially in the liver [47, 104, 105]. In cultured adipocytes, though, metformin blocked isoprenaline- and atrial natriuretic peptide-induced lipolysis [14], and in human tracer studies, it was demonstrated to increase hepatic de novo lipogenesis [40]. Opposing effects of clinical metformin administration on lipid metabolism-related genes have also been noted in skeletal muscle versus adipose tissue [64], suggesting the existence of a futile cycle. Metabolic inefficiency in consequence of metformin treatment has been indicated by the established, albeit modest weight loss in clinical studies [111].

Methionine restriction is a dietary intervention that has been shown to induce profound metabolic alterations in rats [87] and mice [37], resulting in an antidiabetic phenotype [23, 75, 88]. The antidiabetic effect is accompanied by pronounced metabolic inefficiency, as evidenced by about 50% increased energy expenditure and relative food consumption of these animals [44, 88]. Loss of hepatic complex I protein expression after methionine restriction has been shown in rats, even if other complexes were also affected and oxygen consumption rates were unimpaired [22, 93]. In addition, a loss of hepatic complex I enzyme activity has been reported in pigs following methionine restriction 112. Metabolic assays [85], transcriptome analyses [87], and physiological studies [44, 45] have all indicated the occurrence of fatty acid cycling as a consequence of methionine restriction in rodents.

Thiazolidinediones (glitazones) are antidiabetic drugs whose impact on energy metabolism is generally attributed to their activation of the peroxisome proliferator-activated receptor γ (PPARγ) in adipocytes [46]. However, glitazones may also exert relevant metabolic effects independently of this receptor [35]. Pioglitazone, for example, has been reported to act as inhibitor and disassembly factor of complex I [36] at concentrations found in human serum [20]. Similar observations have been made for rosiglitazone [18]. Gene expression analyses indicated that fatty acid synthesis [28] as well as fatty acid oxidation [11, 12] were upregulated following glitazone treatment in humans, potentially reflecting futile fatty acid cycling.

Lipid-lowering drugs of the fibrate class activate the peroxisome proliferator-activated receptor α (PPARα) as their canonic mechanism of action [99]. Notably, they also possess anti-diabetic properties [26] that may become relevant in certain clinical situations [32]. Beyond acting as PPARα agonist, fenofibrate was found to inhibit complex I [19] at clinically encountered concentrations [77]. Concomitantly, fenofibrate has explicitly been shown to induce fatty acid cycling in mice [82].

The paradoxical nature of the induction of an anabolic and energy-demanding metabolic process like fatty acid synthesis in relation to energy-curtailing complex I inhibition prompted us to investigate this phenomenon further. Our studies revealed that the concomitant synthesis and degradation of fatty acids physiologically serves the conversion of NADPH into respirable FADH_2_, which enables NAD^+^-independent glucose oxidation under emergency conditions.

## Methods

All transcriptomic and metabolomic analyses of complex I inhibition presented in this work are based on experiments whose raw data have been published and deposited elsewhere [5, 49, 87]. Three well-established, but etiologically different models of complex I inhibition were chosen for comparative investigation: (i) MPP-treated neurons, (ii) methionine-restricted rats, and (iii) metformin-treated hepatocytes, as detailed in the following.

### MPP-treated neurons

RNA sequencing data were retrieved from an experiment in which in vitro-differentiated LUHMES cells (Lund human mesencephalic cells, ATCC #CRL-2927) were treated with a subtoxic concentration (10 µM) of MPP (1-methyl-4-phenylpyridinium) for 48 h [5]. LUHMES cells are a widely used model to generate human dopaminergic neuronal cells from immortalized fetal precursors for Parkinson’s disease research [63, 96]. MPP is a selective, reversible complex I inhibitor [79, 90] whose toxicity towards vulnerable cells depends on the induction of ROS (reactive oxygen species) production by reduced complex I [8, 66].

The referred RNA sequencing data from in vitro-differentiated LUHMES cells have been deposited in Gene Expression Omnibus (dataset GSE229460 at www.ncbi.nlm.nih.gov/geo) [5]. Alignment was performed to the Ensembl human reference genome GRCh38 with annotations provided by Gencode release 25 using the STAR v.2.5.2b software code with parameters “–outFilterMismatchNmax 2 –outFilterMultimapNmax 10” [29]. SAMtools v.1.5 was adopted to remove secondary alignments, followed by data quality inspection using RSeQC v2.6.4 and dupRadar v.1.8.0. Quantification of reads was done using the Subread 1.5.1 tool “featureCounts” with stranded option “-s 2” [68]. The Bioconductor package DESeq2 v.1.18.1 was used for the assignment of fold changes (FC) and *p* values (Wald test) as described, adopting a cutoff of 1% false discovery rate (FDR) [5, 70]. Unless otherwise stated, all transcripts with an expression level of more than one RPKM (reads per kilobase of transcript per million mapped reads) were included in the analysis. Modulatory effects were considered significant below the *p* < 10^−6^ level (*n* = 3).

### Methionine-restricted rats

Transcriptomic and metabolomic data characterizing methionine restriction were collected from an in vivo experiment in male F344 rats [87], in which 6-week-old animals were assigned to a methionine-restricted diet containing 0.17% methionine or a control diet containing 0.86% methionine for 3 months. Dietary methionine restriction has profound metabolic effects that elicit a robustly antidiabetic and weight loss phenotype [44, 88]. Different experimental protocols have provided evidence for a loss of hepatic complex I expression after methionine restriction in Wistar rats [22, 93] and crossbred piglets 112.

In the referred study [87], tissue samples from the liver, quadriceps muscle, and inguinal adipose tissue of methionine-restricted rats were analyzed for changes in gene expression using microarrays. The microarray data were processed with GeneSpring GX 11 using PLIER16 and noise-filtered. Significant changes were identified by ANOVA with Benjamini–Hochberg correction. All data fulfilling two thresholds, *p* < 0.05 and FC > 3/2 (or FC < 2/3), were included in the current analysis in the exact same manner as in the original study [87].

The liver, quadriceps muscle, inguinal adipose tissue and serum were also analyzed for metabolomic alterations by two UHPLC/MS/MS (ultra-high performance liquid chromatography/tandem mass spectrometry) platforms optimized for the detection of acidic and basic species, respectively, and by GC/MS to detect volatile and hydrophobic metabolites [87]. Metabolite identification and quantification were done with custom-made software developed by Metabolon (Durham, NC, USA), the company that performed the analyses. Statistical analysis was done by ANOVA.

### Metformin-treated hepatocytes

Multiplex RNA sequencing data were obtained from an experiment in which HepG2 cells (human hepatocellular carcinoma cells, ATCC #HB-8065) were treated with the biguanide drug metformin at a concentration of 3 mM for 12 h [49]. HepG2 cells are the most widely studied hepatic tumor cell line as they recapitulate various characteristics of mature hepatocytes [4], including comparable metabolic and secretory profiles [4, 55]. Metformin is a classic antidiabetic drug [91, 92] and atypical complex I inhibitor that appears to uncouple the electron transfer and proton pumping activities of complex I, thus exhibiting moderate potential to induce ROS formation [16, 21]. The structural basis of complex I inhibition by metformin has recently been elucidated [17].

The referred RNA sequencing data of metformin-treated HepG2 cells have been published [49]. The associated raw data were obtained from the SRA Run Archive (Bioproject PRJNA858837 at www.ncbi.nlm.nih.gov/sra). Alignment was performed to the Ensembl human genome 42/GRCh38 with the corresponding Ensembl annotation using STAR 2.7.0a [29]. For genome generation, the parameter “–sjdbOverhang” was adjusted to the read lengths of the respective experiments. Mapping was performed using the parameters “–outFilterMismatchNmax 2 –outFilterMultimapNmax 10 –outSAMattributes All –outSAMtype BAM SortedByCoordinate –outReadsUnmapped Fastx –outMultimapperOrder Random –outWigType wiggle.” Quantification was done with the Subread 1.6.3 function “featureCounts” [68]. Finally, count data were analyzed using DESeq2 v.1.36.0 [70]. As in the MPP experiment, all robustly expressed transcripts with more than one RPKM were considered for analysis, adopting a significance threshold of *p* < 10^−6^ (*n* = 3).

### Gene selection

Gene lists were manually assembled for all catalytic proteins being canonically involved in the following metabolic pathways: glycolysis, citric acid cycle, pentose phosphate pathway, citrate shuttle, fatty acid synthesis, carnitine shuttle, and fatty acid oxidation. Metabolic pathways were defined as logical assemblies of catalytically active enzymes in accordance with the Reactome database, release 82 or later [38]. The expression of merely regulatory factors such as regulatory kinases was excluded from the analysis because of their often unknown or pronouncedly non-linear contribution to metabolic flux. Substrate transporters were systematically sampled only for the analysis of methionine-restricted rats, which exhibited a smaller number of regulatory changes in general. Regarding both RNA sequencing experiments, only those metabolite transporters were investigated that are canonically involved in two transmembrane metabolic cycles, specifically, the carnitine shuttle and the citrate shuttle. All considered genes as proposed by the Reactome database were also inspected manually to comply with the basic logic of metabolism. No group analyses by keyword or clusterings of any type were performed.

## Results

### *Fatty acid cycling produces a metabolic conversion of NADPH into FADH*_*2*_

**Fig. 1:**
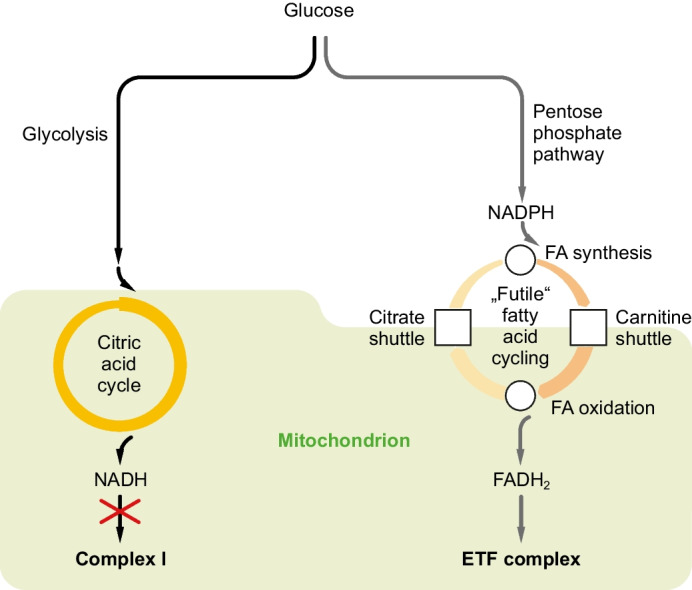
NAD^+^-independent glucose oxidation. Complex I inhibition blocks the default pathway of glucose oxidation via glycolysis and the citric acid cycle. Consequently, intracellular glucose-6-phosphate is diverted to the pentose phosphate pathway, whose NADPH output is translated into FADH_2_ by fatty acid cycling. The obtained FADH_2_ can then be used to fuel the respiratory chain via the electron-transferring flavoprotein (ETF) complex

Fatty acid cycling is an ostensibly energy-wasting process that is usually considered futile when focusing exclusively on ATP generation. To more closely define the degree of futility of this metabolic phenomenon, the individual enzymatic steps and cofactors required for one round of substrate-neutral fatty acid cycling were assembled (Table 1—II). Fatty acid cycling was defined here as the concomitant or consecutive synthesis of palmitoyl-CoA from acetyl-CoA by the cytosolic fatty acid synthase complex, and the degradation of the newly produced palmitoyl-CoA to acetyl-CoA by the enzymes of mitochondrial β-oxidation. More specifically, a complete round of fatty acid cycling in this sense would involve the consecutive action of four discrete metabolic processes (Fig. 1).

**Fig. 2:**
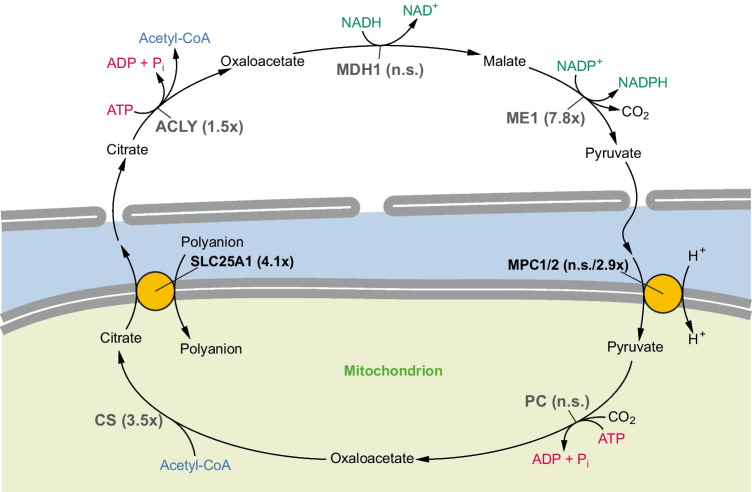
Gene expression changes of citrate shuttle enzymes after complex I inhibition by MPP in vitro. The depicted circuitry of the citrate shuttle represents its most widely discussed variant involving cytosolic oxaloacetate recycling as pyruvate. The mitochondrial citrate transporter SLC25A1 accepts different polyanions as exchange substrates, canonically malate (to be recycled to the cytosol by SLC25A10 in exchange for phosphate; not shown), but also phosphoenolpyruvate (PEP), succinate, and isocitrate [74]. PEP may play a primary role in the export of citrate after complex I inhibition because its import does not involve the accidental co-import of reducing equivalents into the already NADH-overloaded mitochondrion. Abbreviations are used as in Table 2. Expression fold changes after MPP treatment are given in brackets; n.s., not significant

(i) *Fatty acid synthesis:* the reductive coupling of cytosolic acetyl-CoA to a growing fatty acid chain by fatty acid synthase (FASN) requires the prior activation of the acetyl-CoA molecule to malonyl-CoA by acetyl-CoA carboxylase α (ACACA). The latter reaction consumes one molecule of ATP, whereas the reductive steps of the FASN reaction consume two molecules of NADPH per acetyl-CoA.
(ii) *Carnitine shuttle:* palmitoyl-CoA targeted for β-oxidation needs to be imported into the mitochondrion via the carnitine shuttle system. This system works cofactor-neutral, except for a negligible energetic consumption associated with the mitochondrial import of CoA in exchange for ADP via SLC25A42.
(iii) *Fatty acid oxidation:* per acetyl-CoA released, mitochondrial β-oxidation provides one molecule of NADH by hydroxyacyl-CoA dehydrogenase (HADH), and one molecule of FADH_2_ by acyl-CoA dehydrogenase (ACAD).
(iv) *Citrate shuttle:* to complete the cycle, mitochondrial acetyl-CoA from β-oxidation needs to be exported by the citrate shuttle system. In its most widely considered variant, which includes the action of malic enzyme [41, 62], this energetically demanding shuttle consumes two molecules of ATP per acetyl-CoA exported: one at the ATP citrate lyase (ACLY) step, and one at the pyruvate carboxylase (PC) step (Fig. 2). Moreover, it consumes one molecule of NADH by malate dehydrogenase (MDH), and it generates one molecule of NADPH by malic enzyme (ME).

**Table 1:** Cofactor balance of the inferred pathway of complex I-independent glucose oxidation (“NADPH-FADH_2_ axis”)

**I—Pentose phosphate pathway** * Yield per glucose:*			
	ATP + 12 NADP	→	ADP + 12 NADPH
**II—Fatty acid cycling** * Yield per acetyl-CoA:*			
FA synthesis	ATP + 2 NADPH	→	ADP + 2 NADP
Carnitine shuttle	—	→	—
FA oxidation	NAD^+^ + FAD	→	NADH + FADH_2_
Citrate shuttle	2 ATP + NADH + NADP	→	2 ADP + NAD^+^ + NADPH
Sum:	3 ATP + NADPH + FAD	→	3 ADP + NADP + FADH_2_
**III—Total of I + II** * Yield per glucose (1* × *pentose phosphate pathway* + *12* × *fatty acid cycling):*			
	37 ATP + 12 FAD	→	37 ADP + 12 FADH_2_
Overall energetic outcome: -19 ATP per glucose*			
**IV—Comparison with glycolysis/citric acid cycle** * Yield per glucose (ATP* ~ *GTP):*			
	4 ADP + 10 NAD^+^ + 2 FAD	→	4 ATP + 10 NADH + 2 FADH_2_
Overall energetic outcome: +32 ATP per glucose*			

^*^Based on the assumption that the oxidation of NADH will provide 2.5 ATP, and that the oxidation of FADH_2_ will provide 1.5 ATP

Calculating the cofactor balance of the above reactions that would be associated with the complete turnover of one molecule of acetyl-CoA, the final result was a loss of three ATP, the oxidation of one NADPH, and the reduction of one FAD (Table 1). In other words, fatty acid cycling does not only consume ATP as expected, but it concomitantly translates the anabolic, non-respirable cofactor NADPH into the respirable, catabolic cofactor FADH_2_. FADH_2_ provides electrons to the respiratory chain via the electron-transferring flavoprotein (ETF) complex [48], which is still operable under conditions of complex I blockade. Hence, the 1:1 conversion of NADPH into FADH_2_ by fatty acid cycling offers a metabolic means of entirely NAD^+^-independent glucose oxidation through linking up the NADPH-producing pentose phosphate pathway with fatty acid cycling and FADH_2_ oxidation (Fig. 1).

The energetic outcome of this linkage after stoichiometric adjustment, i.e., when NADPH production and consumption are equal, was determined as follows (Table 1): the complete oxidation of one molecule of glucose to carbon dioxide costs one ATP and yields 12 NADPH, which can be 1:1 translated into 12 respirable FADH_2_ by the cycling of 12 acetyl-CoA units consuming 36 ATP. Oxidation of 12 FADH_2_ in turn provides approximately 18 ATP, which only partially compensates for the loss caused by fatty acid cycling. The final score of the here described “NADPH-FADH_2_ axis” of complete glucose oxidation via the pentose phosphate pathway and subsequent fatty acid cycling is a deficit of − 19 ATP per glucose. For comparison, the standard pathway of complete glucose oxidation via glycolysis and the citric acid cycle yields + 32 ATP per glucose (Table 1).

Hence, the oxidation of glucose via the NADPH-FADH_2_ axis cannot constitute a sustainable way of energy generation in whole organisms for extended periods of time. Nevertheless, as described in the introduction, fatty acid cycling appears to arise frequently in vivo and under different pharmacological and physiological conditions. Embedded in the context of adverse complex I inhibition, the latter observation might find a straightforward explanation: when complex I inhibition curtails the citric acid cycle and glycolysis through an increase in the NADH/NAD^+^ ratio, an increase in cytosolic citrate and other mechanisms [2, 52], then the induction of the NADPH-FADH_2_ axis will represent a beneficial and potentially cytoprotective response. In such a case, the NADPH-FADH_2_ axis will prevent cellular glucose overload [56] and will maintain mitochondrial membrane potential and ATP levels in more severely affected, complex I-inhibited organs (e.g., the liver) at the energetic cost of other, less affected organs (e.g., adipose tissue).

To explore whether the NADPH-FADH_2_ axis was indeed induced by complex I inhibition at the cellular or animal level, we have reanalyzed the published transcriptomic datasets of three different models of complex I inhibition, namely MPP-treated LUHMES neurons [5], methionine-restricted F344 rats [87], and metformin-treated HepG2 hepatocytes [49].

### *Evidence for the NADPH-FADH*_*2*_* axis from MPP-treated neuronal cells*

MPP (1-methyl-4-phenylpyridinium) is a selective complex I inhibitor [79, 90] widely employed to induce dopaminergic cell death modeling Parkinson’s disease [8, 80]. In the reanalyzed RNA sequencing experiment, subtoxic doses of MPP were administered to in vitro-differentiated neuronal human cells (LUHMES cells) for 48 h [5]. Under these experimental conditions, ATP levels were unaltered by the drug, whereas the cellular NADH/NAD^+^ ratio and the concentration of lactate in the medium were significantly increased [5]. Of the 58,038 RNAs detected by sequencing, 10,924 belonged to genes expressed at a level of more than one RPKM. Of these genes, 6034 were significantly modulated at the *p* < 10^−6^ level (*n* = 3), including 2898 upregulated and 3136 downregulated genes [5].

Surveying the changes in metabolic gene expression induced by complex I inhibition in LUHMES cells, a systematic and strong induction of glycolytic genes was found, accompanied by a similarly pronounced and almost unanimous upregulation of citric acid cycle genes (Table 2). The increased expression of these genes could be plausibly interpreted as a direct compensatory response to the overall inhibition of the glycolysis/citric acid cycle/respiratory chain axis by complex I inhibition. At the same time, there was a significant upregulation of genes encoding key enzymes of the pentose phosphate pathway, which is generally regarded as a secondary pathway of glucose oxidation that is committed to the provision of NADPH for anabolic purposes of growth and repair [101]. Specifically, the genes for both dehydrogenases of the pentose phosphate pathway, glucose-6-phosphate dehydrogenase (G6PD) and phosphogluconate dehydrogenase (PGD), were significantly induced (1.8-fold and 3.2-fold, respectively) (Table 2), as was the gene for transketolase (8.6-fold), the enzyme that is generally rate-limiting for the non-oxidative branch of this pathway [94]. Since the G6PD and PGD genes were already expressed highly at baseline, i.e., higher than any citric acid cycle enzyme (Table 2), the expression changes of these rate-limiting dehydrogenases [101, 110] suggest a substantially increased capacity of NADPH formation in the MPP-treated LUHMES cells. The concept of a purposefully pursued investment in NADPH formation capacity after complex I inhibition is supported by the observation that out of ten metabolic sources of NADPH listed in an authoritative review article [110], nine were significantly induced (between 1.4-fold and 7.8-fold) (Suppl. Tab. 1).

**Table 2:** Gene expression after complex I inhibition by MPP in LUHMES cells

Gene	Fold change	*p* value	RPKM baseline	Gene name
Glycolysis				
GCK	3.16	1E − 31	2	Glucokinase
HK1	2.49	4E − 33	29	Hexokinase 1
HK2	3.72	6E − 72	3	Hexokinase 2
GPI	3.25	3E − 48	13	Glucose-6-phosphate isomerase
PFKM	1.89	6E − 14	24	Phosphofructokinase, muscle
ALDOA	3.92	4E − 62	45	Fructose-bisphosphate aldolase A
ALDOC	4.23	5E − 75	11	Fructose-bisphosphate aldolase C
TPI1	2.82	1E − 37	264	Triosephosphate isomerase 1
GAPDH	2.93	3E − 36	642	Glyceraldehyde-3-phosphate dehydrogenase
PGAM2	2.82	3E − 08	2	Phosphoglycerate mutase 2 (muscle)
ENO1	2.17	4E − 25	157	Enolase 1
ENO2	2.38	5E − 35	30	Enolase 2
ENO3	5.03	9E − 105	31	Enolase 3
PKM	1.49	5E − 07	91	Pyruvate kinase M1/2
Citric acid cycle				
CS	3.53	4E − 65	31	Citrate synthase
ACO2	1.95	3E − 17	33	Aconitase 2
IDH1	1.40	7E − 07	53	Isocitrate dehydrogenase 1 (NADP +)
IDH2	1.93	9E − 17	23	Isocitrate dehydrogenase 2 (NADP +)
IDH3B	1.93	4E − 18	28	Isocitrate dehydrogenase 3 (NAD +) non-catalytic subunit β
IDH3G	1.67	1E − 08	13	Isocitrate dehydrogenase 3 (NAD +) non-catalytic subunit γ
DLD	0.53	5E − 14	19	Dihydrolipoamide dehydrogenase
SUCLG1	3.14	4E − 47	18	Succinate-CoA ligase subunit α
SUCLG2	2.01	1E − 08	2	Succinate-CoA ligase [GDP-forming] subunit β
SDHA	1.73	7E − 17	10	Succinate dehydrogenase complex flavoprotein subunit A
SDHB	1.80	2E − 13	23	Succinate dehydrogenase complex iron sulfur subunit B
FH	2.81	5E − 36	25	Fumarate hydratase
MDH2	2.71	4E − 36	44	Malate dehydrogenase 2, mitochondrial
Pentose phosphate pathway				
G6PD	1.75	9E − 14	56	Glucose-6-phosphate dehydrogenase
PGLS	2.13	1E − 14	11	6-Phosphogluconolactonase
PGD	3.16	3E − 45	117	Phosphogluconate dehydrogenase
TKT	8.63	4E − 153	24	Transketolase
Citrate shuttle				
CS	3.53	4E − 65	31	Citrate synthase
SLC25A1	4.11	9E − 78	16	Solute carrier family 25 member 1
ACLY	1.47	2E − 09	146	ATP citrate lyase
MDH2	2.72	4E − 36	44	Malate dehydrogenase 2, mitochondrial
ME1	7.78	2E − 97	1	Malic enzyme 1, cytosolic
ME2	1.79	3E − 18	7	Malic enzyme 2, mitochondrial
MPC2	2.89	4E − 31	7	Mitochondrial pyruvate carrier 2
Fatty acid synthesis				
ACACA	2.77	1E − 53	11	Acetyl-CoA carboxylase α
ACSL1	0.44	3E − 26	12	Long-chain-fatty-acid—CoA ligase 1
ACSL3	2.07	8E − 11	18	Long-chain-fatty-acid—CoA ligase 3
ACSL4	1.92	7E − 11	12	Long-chain-fatty-acid—CoA ligase 4
ELOVL1	2.10	2E − 18	5	ELOVL fatty acid elongase 1
ELOVL5	2.69	4E − 57	35	ELOVL fatty acid elongase 5
ELOVL6	1.69	8E − 08	11	ELOVL fatty acid elongase 6
HACD2	0.46	2E − 14	3	Very-long-chain 3-hydroxyacyl-CoA dehydratase 2
TECR	1.68	3E − 11	14	Trans-2,3-enoyl-CoA reductase
SCD	7.78	8E − 255	160	Stearoyl-CoA desaturase
SCD5	1.73	1E − 12	6	Stearoyl-CoA desaturase 5
Carnitine shuttle (n.a.)				
Fatty acid oxidation				
ACADM	0.40	1E − 18	13	Acyl-CoA dehydrogenase, medium chain
ACADVL	2.16	9E − 23	20	Acyl-CoA dehydrogenase very long chain
ACAD9	0.50	1E − 13	8	Acyl-CoA dehydrogenase family member 9
HADH	4.14	1E − 67	6	Hydroxyacyl-CoA dehydrogenase
HADHA	1.69	9E − 15	53	Trifunctional enzyme subunit α
HADHB	1.99	7E − 23	20	Trifunctional enzyme subunit β

Sampling of all significantly induced or repressed genes (*p* < 10^−6^; *n* = 3) with robust baseline expression (RPKM > 1) for the following metabolic pathways: glycolysis, citric acid cycle, pentose phosphate pathway, citrate shuttle, fatty acid synthesis, carnitine shuttle, and fatty acid oxidation. A complete list of genes irrespective of their *p* value is provided in Suppl. Tab. 1, including a sampling of all detected NADPH-generating enzymes. *n.a.*, not applicable (no significant changes occurred)

Beyond NADPH formation, other key elements of the NADPH-FADH_2_ axis were coherently upregulated in complex I-inhibited neuronal cells. Fatty acid biosynthesis requires the coordinated cytosolic delivery of NADPH as well as acetyl-CoA units either stemming from glycolysis and the mitochondrial pyruvate dehydrogenase complex (under normal conditions), or stemming from mitochondrial β-oxidation (during “futile” fatty acid cycling). In both cases, mitochondrially produced acetyl-CoA is exported to the cytosol via the energetically costly citrate shuttle system. As listed in Table 2 and illustrated in Fig. 2, the genes encoding the signature enzymes of the citrate shuttle were all induced by complex I inhibition, specifically mitochondrial citrate synthase (CS) (3.5-fold), the mitochondrial citrate transporter SLC25A1 (4.1-fold), and cytosolic ATP citrate lyase (ACLY) (1.5-fold). Several additional genes encoding enzymes potentially involved in this multi-branched shuttle system [41, 62] were also transcriptionally upregulated (Table 2).

Considering the fatty acid biosynthesis pathway, a pronounced induction of genes encoding for several key enzymes was evident. Specifically, the rate-limiting enzyme acetyl-CoA carboxylase α (ACACA) was induced 2.8-fold at the mRNA level, as were the highly expressed marker enzyme stearoyl-CoA desaturase (SCD) (7.8-fold) and different fatty acid elongases (ELOVL1, ELOVL5, and ELOVL6) (Table 2). On the other hand, a heterogeneous gene expression pattern was observed for the long-chain acyl-CoA synthetase family, in which ACSL1 was downregulated, whereas ACSL3 and ACSL4 were upregulated. In view of the closely overlapping functions of these enzymes [61], the origin of this observation is unclear. The lack of modulation of the highly expressed household gene for the multi-enzyme fatty acid synthase (FASN) in the current neuronal cell model may be related to its previously reported, global resistance to transcriptional regulation in tissues other than the liver and adipose tissue [25]. Instead, fatty acid biosynthetic flux is predominantly controlled by the activity of acetyl-CoA carboxylase α (ACACA), which in turn is regulated by polymerization triggered by the MID1-interacting protein 1 (MID1IP1) [60]. In fact, the gene for MID1IP1 was strongly induced in the MPP-treated LUHMES cells (5.3-fold) (Table 3). Subsequently, internal allosteric activation of FASN by accumulating acyl carrier protein (ACP)-bound substrates may occur, as structurally demonstrated [102], and may represent the main mode of regulation of FASN. Altogether, the conclusion is warranted that MPP-treated LUHMES cells engage in enhanced NADPH-dependent fatty acid biosynthesis.

**Table 3:** Expression of selected regulatory genes of fatty acid metabolism in three models of complex I inhibition

Gene	MPP		MetR (liver)		MetR (adipose)		Metformin		Mode of action
	FC	*p* value	FC	*p* value	FC	*p* value	FC	*p* value	
BRCA1	**[0.29]**	1E − 10	—	—	—	—	**0.16**	2E − 56	Inhibition of ACACA
MID1IP1	**5.30**	1E − 67	—	—	—	—	**2.15**	5E − 87	Activation of ACACA
ACACB	**[1.77]**	5E − 08	**0.32**	< 0.001	0.86	0.7	**0.30**	7E − 34	Inhibition of β-oxidation
CD36	[0.71]	0.7	**8.30**	< 0.001	—	—	[0.56]	0.02	Fatty acid uptake
LDLR	**5.33**	< 1E − 99	—	—	—	—	**2.89**	< 1E − 99	Cholesterol and lipid uptake
VLDLR	1.17	0.1	**92.78**	< 0.001	—	—	**[0.43]**	1E − 23	Triglyceride uptake
FGF21	[57.74]	2E − 04	**16.07**	< 0.001	**1.84**	0.003	[5.61]	0.2	Induction of lipolysis
GDF15	**[7.42]**	7E − 11	—	—	—	—	**9.65**	< 1E − 99	Induction of lipolysis

Data for MPP- and metformin-treated cells were derived from RNA sequencing experiments, whereas data for methionine-restricted rats were obtained from manually performed RT-PCR experiments. Statistically significant changes are highlighted in bold; different significance thresholds were applicable in the different experiments as described in the “Methods” section. Brackets denote that the corresponding gene had a low expression level (RPKM < 1), warranting cautious interpretation. FC, fold change; MetR, methionine restriction; —, not available

Looking at the transcriptional changes related to mitochondrial fatty acid uptake and oxidation, nine genes were expressed above the adopted threshold of one RPKM (Table 2; Suppl. Tab. 1). Of these, seven were numerically, and four were significantly induced after MPP treatment. Among the latter genes were both chains of the α_2_β_2_-tetrameric trifunctional enzyme (HADHA: 1.7-fold; HADHB: 2.0-fold) as well as the very long chain acyl-CoA dehydrogenase (ACADVL, 2.2-fold), which is functionally associated with the trifunctional enzyme and initiates the oxidation of C_12_-C_24_ fatty acids [6, 109]. The gene for medium chain acyl-CoA dehydrogenase (ACADM), which primarily oxidizes C_4_-C_12_ fatty acids, was downregulated, however (0.40-fold), whereas the gene for the hydroxyacyl-CoA dehydrogenase with the same chain length preference as ACADM was highly induced (HADH, 4.1-fold). Acyl-CoA dehydrogenase 9 (ACAD9), a multifunctional complex I chaperone and likely metabolic dehydrogenase [98], was also downregulated (0.50-fold). Carnitine shuttle enzymes were unaltered. The remarkable induction of the full set of genes necessary for very long chain fatty acid oxidation in parallel with the induction of genes encoding the enzymes for malonyl-CoA production, stearoyl-CoA desaturation, and fatty acid elongation suggests that fatty acid cycling occurs in the current model and is primarily conducted with very long chain fatty acids.

In summary, all major elements of the NADPH-FADH_2_ axis were induced at the mRNA level by complex I inhibition in LUHMES cells. The degree of induction of the NADPH-FADH_2_ axis was similar to the degree of induction of glycolysis and the citric acid cycle, the expected default compensatory response to complex I inhibition.

### *Evidence for the NADPH-FADH*_*2*_* axis from methionine-restricted rats*

Methionine restriction is a defined nutritional intervention that increases life span in various animal species, including mice and rats [78, 83, 88]. In parallel, methionine restriction causes severe metabolic inefficiency, restricts weight gain, and induces a robust antidiabetic phenotype [23, 75, 88]. Despite a modest, arguably compensatory induction of mitochondrial biogenesis during methionine restriction [86], a pronounced loss of complex I protein expression and activity has been reported [22, 93, 112]. The molecular origin of the latter observation is unknown at present. Whether the loss of complex I is related to the exceedingly high methionine content of this protein complex [10, 95] remains to be determined.

In the reanalyzed microarray and metabolomics study of 3-month methionine-restricted rats [87], the adopted significance threshold of *p* < 0.05 (*n* = 6) and an effect size (fold change) threshold of FC > 3/2 (or FC < 2/3) identified 285 regulated genes in liver (172 up and 113 down), 224 regulated genes in adipose tissue (123 up and 101 down), and 80 regulated genes in skeletal muscle (39 up and 41 down) [87]. In the metabolomics analyses, 444 biochemicals were detected in the liver, of which 65 were increased, and 164 were decreased when adopting a *p* < 0.05 (*n* = 5) significance threshold. The corresponding numbers of biochemicals in the other tissues were as follows: 248 in adipose tissue (30 increased, 9 decreased), 362 in skeletal muscle (33 increased, 51 decreased), and 332 in serum (55 increased, 86 decreased) [87].

**Fig. 3:**
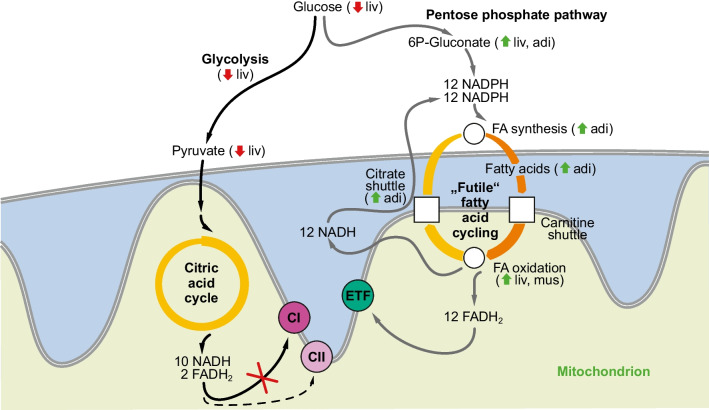
Tissue-integrating sketch of key changes in gene expression and metabolite concentrations induced by methionine restriction in vivo. Methionine restriction leads to a systematic shift away from glycolysis towards fatty acid uptake and β-oxidation in the liver (liv). In adipose tissue (adi), there is a complementary induction of glucose degradation, fatty acid synthesis and fatty acid provision for export. Fatty acid uptake and oxidation are also induced in skeletal muscle (mus). In case of an incomplete inhibition of complex I in the liver, both metabolic axes (left/right) may be operable in parallel

With regard to the selected repertoire of metabolic pathways that were analyzed in this study, a complete survey of the significantly altered mRNAs in liver, adipose tissue, and skeletal muscle is provided in Table 4, and a selection of key metabolites detected in the same tissues is listed in Table 5. In comparison with cultured LUHMES cells, only a smaller number of genes were significantly altered in this in vivo model. Nevertheless, a rather clear pattern emerged (Fig. 3), indicating that fatty acid cycling indeed occurred in the methionine-restricted rats, but that it was compartmentalized between two tissues, namely the liver and adipose tissue. Adipose tissue showed a signature induction of genes for fatty acid biosynthesis and provision to other tissues, including acetyl-CoA carboxylase α (ACACA) (3.4-fold), stearoyl-CoA desaturase (SCD) (2.7-fold), hormone-sensitive lipase (LIPE) (1.7-fold), and monoacylglycerol lipase (MGLL) (1.6-fold). Corresponding with the gene expression signature, free fatty acids were unanimously and often significantly elevated in adipose tissue (Table 5). The direction of flow of the synthesized or mobilized fatty acids was apparent from the observed concentration gradient: serum fatty acids were mildly, but not significantly elevated, whereas in the liver, fatty acid concentrations were significantly decreased (Table 5). The inferred demand of the liver for fatty acids as major energy source (instead of glucose) was further indicated by the significantly increased gene expression of fatty acid translocase (CD36) (1.8-fold) and very low-density lipoprotein receptors (VLDLR) (5.8-fold). These microarray data were validated by TaqMan quantitative PCR, which specified that the expression of the CD36 gene was induced by 8.3-fold, and the VLDLR gene was induced by 93-fold (Table 3). A pronounced depletion of plasma triglycerides of about 65% in methionine-restricted rats has been noted before [45, 88], underscoring the likely functionality of the VLDLR mRNA increase. The observed transcriptional boost of the hepatic fat hunger signal fibroblast growth factor 21 (FGF21) (16-fold) also supports the above conclusion (Table 3) 50. In the liver, increased β-oxidation was suggested by the mRNA induction of acyl-CoA thioesterase 2 (ACOT2) (1.7-fold) and the trifunctional enzyme subunit β (HADHB) (1.6-fold), but perhaps even more so from the transcriptional suppression of acetyl-CoA carboxylase β (ACACB) (0.46-fold), a key negative regulator of β-oxidation [1]. Finally, increased hepatic fatty acid utilization was evidenced by the substantially raised levels of the signature ketone β-hydroxybutyrate in plasma (2.4-fold).

**Table 4:** Gene expression changes following methionine restriction in rats

	Liver			Adipose			Muscle		
	Gene	FC	*p* value	Gene	FC	*p* value	Gene	FC	*p* value
Glycolysis and glucose uptake	GCK	**0.55**	9E − 04	*LDHA*	**1.74**	2E − 03	*LDHB*	**2.58**	3E − 02
	PKLR	**0.62**	2E − 03	PFKM	**1.65**	4E − 02			
				SLC2A4	**1.89**	8E − 04			
Citric acid cycle	*ACO2*	**1.57**	3E − 07	*ACO2*	**1.56**	9E − 04			
				IDH3A	**1.76**	1E − 04			
				MDH2	**1.57**	3E − 04			
Pentose phosphate pathway and NADPH provision	ME1	**1.70**	6E − 03						
	MTHFD2	**4.54**	1E − 06						
Citrate shuttle	*ACLY*	**0.48**	1E − 03	*ACLY*	**1.68**	1E − 02			
	ME1	**1.70**	6E − 03	MDH1	**1.57**	9E − 04			
				PC	**1.71**	1E − 03			
Fatty acid synthesis and provision	*ACACB*	**0.46**	2E − 07	*ACACA*	**3.35**	3E − 06			
	*ELOVL5*	**0.65**	3E − 05	*ELOVL6*	**4.04**	3E − 05	*ELOVL6*	**2.04**	5E − 02
	*SCD*	**0.07**	5E − 06	*SCD*	**2.66**	2E − 04			
				*ACSL1*	**1.72**	4E − 03	*ACSL3*	**0.58**	7E − 03
				HSD17B12	**1.79**	5E − 05			
				*LIPE*	**1.71**	1E − 02			
				MGLL	**1.61**	2E − 02			
Carnitine shuttle				CPT1B	**1.80**	1E − 03			
Fatty acid oxidation and uptake	*CD36*	**1.78**	6E − 04	APOBR	**0.62**	1E − 02	*CD36*	**1.71**	3E − 02
	ACOT2	**1.66**	1E − 05				ACAA2	**1.55**	1E − 02
	HADHB	**1.62**	5E − 04				ACSF2	**1.52**	4E − 03
	VLDLR	**5.79**	2E − 09				*LIPE*	**1.68**	6E − 03
							LPL	**1.75**	2E − 02

Complete sampling of all significantly (*p* < 0.05) and parametrically (FC > 3/2 or FC < 2/3) altered metabolic genes in three tissues (liver, inguinal adipose tissue, quadriceps muscle) of methionine-restricted rats as per microarray experiment (*n* = 6). The following metabolic pathways were sampled: glycolysis and glucose uptake, citric acid cycle, pentose phosphate pathway and NADPH provision, citrate shuttle, fatty acid synthesis and provision, carnitine shuttle, and fatty acid oxidation and uptake. Italics indicate the significant modulation of a gene or one of its paralogues in more than one tissue. Hormone-sensitive lipase (LIPE) has divergent functions in adipose tissue (provision of fatty acids for export) and skeletal muscle (provision of fatty acids for local oxidation). Gene names are used as in Table 2 and Suppl. Tab. 1; additional genes were: LDHA/B, lactate dehydrogenase A/B; PKLR, pyruvate kinase L/R; SLC2A4, facilitated glucose transporter member 4 (GLUT4); LIPE, lipase E, hormone-sensitive; MGLL, monoglyceride lipase; CD36, fatty acid translocase; APOBR, apolipoprotein B receptor; ACOT2, acyl-CoA thioesterase 2; ACAA2, acetyl-CoA acyltransferase 2; ACSF2, acyl-CoA synthetase family member 2; MTHFD2, methylenetetrahydrofolate dehydrogenase/cyclohydrolase; VLDLR, very low-density lipoprotein receptor; LPL, lipoprotein lipase. FC, fold change

**Table 5:** Metabolite changes following methionine restriction in rats

		Liver	Adipose	Muscle	Serum
		FC	FC	FC	FC
Glycolytic intermediates	Glucose	**0.80**	0.72	1.14	0.79
	Glucose-6P	—	1.02	4.10	—
	3P-Glycerate	**0.69**	1.35	1.20	—
	PEP	**0.65**	2.80	1.16	—
	Pyruvate	**0.43**	—	1.11	**0.24**
	Lactate	**0.70**	0.88	0.96	0.89
	L/P ratio	**1.63**	—	0.86	**3.71**
Citric acid cycle intermediates	Citrate	—	0.58	**0.53**	**1.26**
	Succinate	1.61	0.72	—	0.90
	Fumarate	0.88	1.29	1.05	0.78
	Malate	0.89	1.13	1.44	0.96
Pentose phosphate pathway intermediates	6P-Gluconate	**1.88**	1.42	—	—
Fatty acids	Palmitate	0.85	1.38	0.93	1.19
	Palmitoleate	**0.52**	**2.37**	0.91	1.46
	Stearate	**0.88**	—	1.06	1.10
	Oleate	0.73	**2.60**	0.76	1.17
	Linoleate	1.01	1.57	0.83	1.29
	Linolenate	1.87	1.47	0.75	1.52
	Arachidonate	1.03	1.40	0.71	0.97
	Docosahexaenoate	0.80	**2.94**	1.78	1.13
Ketone bodies	β-Hydroxybutyrate	1.17	—	**3.22**	**2.44**

Sampling of selected signature metabolites in three tissues (liver, inguinal adipose tissue, quadriceps muscle) and serum of methionine-restricted rats as determined by UHPLC/MS/MS or GC/MS. Significantly altered fold changes (FCs) at the *p* < 0.05 level are highlighted in bold type (*n* = 5). —, not detected in a particular tissue

A concerted metabolite flux between the liver and adipose tissue was further denoted by the transcriptional suppression of the citrate shuttle enzyme ATP citrate lyase (ACLY) in the liver (0.48-fold), as opposed to its induction (1.7-fold) in adipose tissue, and by the hepatic suppression of stearoyl-CoA desaturase (SCD) (0.07-fold), as opposed to its induction (2.7-fold) in adipose tissue. Related methionine restriction experiments in rats have confirmed the pronounced suppression of fatty acid biosynthesis in the liver (and its induction in adipose tissue) at the protein level [45]. Moreover, in vitro studies using freshly isolated inguinal white adipose tissue of rats fed the methionine-restricted diet have indicated an increased capacity of this tissue for β-oxidation of fatty acids, potentially indicative of an additional, local fatty acid cycling within the adipose tissue [24, 45].

**Fig. 4:**
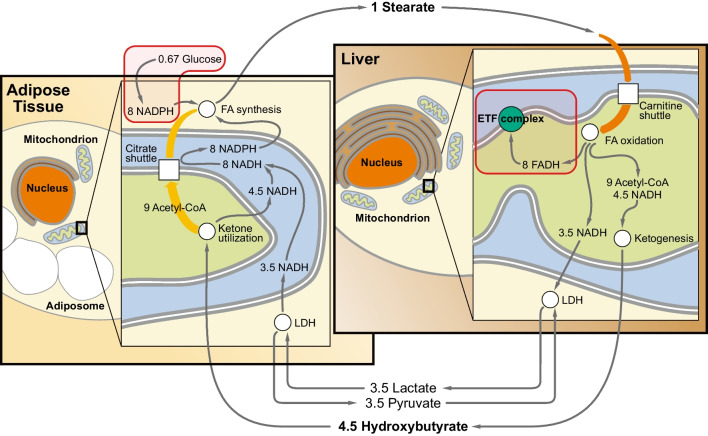
Distribution of fatty acid cycling between the liver and adipose tissue in vivo. Inter-tissue transport processes to maintain fatty acid cycling following hepatic complex I inhibition include the transfer of de novo synthesized fatty acids (e.g., stearate) from adipose tissue to the liver, and the return of acetyl-CoA units from the liver to adipose tissue in the form of ketones (e.g., β-hydroxybutyrate). Complete redox cofactor neutrality would be achieved if the surplus hepatic NADH from β-oxidation were also transported to the adipose tissue via the lactate-pyruvate shuttle as shown. The two core elements of the NADPH-FADH_2_ axis that are connected by fatty acid cycling are boxed in red

Regarding NADPH formation, no individually significant signals were noted except the hepatic induction of the malic enzyme 1 gene (ME1) (1.7-fold) and the methylenetetrahydrofolate dehydrogenase 2 gene (MTHFD2) (4.5-fold). The role of these changes in the liver is unclear, though. Feeding studies in wild-type rats have indicated that cytosolic malic enzyme (ME1) may synthesize NADPH especially for the reductive repair of oxidative damage [100], which is known to be induced by complex I inhibition [8, 80, 103]. On the other hand, the pentose phosphate pathway signature metabolite 6-phosphogluconate was elevated in both the liver and adipose tissue in the presence of lowered glucose levels (Table 5), suggesting fractionally increased flux through this pathway despite unaltered enzyme transcription. Correspondingly, there was a significant reduction of several glycolytic intermediates as well as a reduction of the gene expression of glucokinase (GCK) (0.55-fold) and pyruvate kinase (PKLR) (0.62-fold). Both enzymes are known to exert strong flux control over glycolysis; glucokinase is the prime determinant of glycolytic flux in the working heart under normal conditions, especially when insulin is present [57], whereas pyruvate kinase may be limiting to glycolytic flux in the hypoxic heart, i.e., under conditions of mitochondrial impairment [42]. Together, these changes point towards a switch from glycolytic glucose oxidation to fatty acid uptake and β-oxidation in the liver, whereas the adipose tissue increases its commitment to glucose degradation, as evidenced by the significant induction of the insulin-dependent glucose transporter SLC2A4 (GLUT4) mRNA (1.9-fold). Hence, in the rats fed the methionine-restricted diet, adipose tissue converts glucose into fatty acids, which are subsequently transported to the liver for oxidative degradation and fueling of the respiratory chain via FADH_2_ and the electron-transferring flavoprotein (ETF) (Fig. 4), maintaining respiratory chain function under conditions of complex I insufficiency.

Metabolomic changes in skeletal muscle were largely restricted to the induction of fatty acid uptake and degradation, as based on the increased expression of the fatty acid translocase (CD36) gene (1.7-fold) and the lipoprotein lipase (LPL) gene (1.8-fold). Overall lower levels of fatty acids in muscle, despite the opposite effect in serum, also support the idea that muscle, alongside with the liver, shifted its catabolic activities towards β-oxidation (Table 5), arguably to circumvent the inhibition of complex I.

### *Evidence for the NADPH-FADH*_*2*_* axis from metformin-treated clonal hepatocytes*

Metformin is a widely prescribed antidiabetic drug whose canonic mode of action is an atypical inhibition of mitochondrial complex I [16, 17, 21]. Several other mechanisms such as a glycerophosphate dehydrogenase-mediated suppression of hepatic gluconeogenesis or a direct activation of AMP kinase have been proposed to contribute to the antidiabetic activity of metformin [91]. However, both effects have been challenged experimentally [33, 39] as well as clinically [7, 9] and could also represent downstream sequelae of upstream complex I inhibition [17, 91]. In the reanalyzed RNA sequencing experiment, 3 mM metformin was administered to clonal human hepatocytes (HepG2 cells) for 12 h. In consequence, basal and ATP-coupled mitochondrial respiration were substantially decreased as expected for complex I inhibition [49]. Still, no statistical evidence was found that the transcriptomic changes evoked by metformin were related to AMP kinase activation [49], which would be indicative of a manifest ATP shortage. Out of 62,649 RNAs detected, 11,251 represented genes expressed at a level of more than one RPKM. Of these genes, 5347 were significantly modulated at the *p* < 10^−6^ level (*n* = 3), including 3331 upregulated and 2016 downregulated genes [49].

The induction of the NADPH-FADH_2_ axis by metformin treatment in HepG2 cells was not as conspicuous as in the systems described before. Nevertheless, there was a coherent and significant induction of both signature elements, fatty acid synthesis, and NADPH production (Table 6). For example, the genes of all enzymes of the pentose phosphate pathway were significantly induced after metformin treatment, including glucose-6-phosphate dehydrogenase (G6PD) (1.4-fold), phosphogluconate dehydrogenase (PGD) (1.4-fold), and transketolase (1.6-fold). The only exception was the gene encoding 6-phosphogluconolactonase (PGLS) (0.74-fold), which yet exerts little flux control over the pentose phosphate pathway [101]. The genes of several other NADPH producers were similarly induced, apart from the isocitrate dehydrogenases 1/2 (IDH1/2) (Suppl. Tab. 2).

**Table 6:** Gene expression after complex I inhibition by metformin in HepG2 cells

Gene	Fold change	*p* value	RPKM baseline	Gene name
Glycolysis				
GPI	0.71	4E − 26	51	Glucose-6-phosphate isomerase
ALDOC	0.43	4E − 115	64	Fructose-bisphosphate aldolase C
TPI1	0.61	6E − 63	400	Triosephosphate isomerase 1
PGK1	0.73	2E − 23	179	Phosphoglycerate kinase 1
PGAM1	0.46	2E − 87	90	Phosphoglycerate mutase 1
ENO1	0.64	3E − 57	760	Enolase 1
ENO2	0.55	2E − 46	27	Enolase 2
ENO3	0.55	2E − 19	9	Enolase 3
PKLR	0.61	2E − 30	23	Pyruvate kinase, liver
PKM	0.61	2E − 62	167	Pyruvate kinase M1/2
Citric acid cycle				
CS	1.18	5E − 07	45	Citrate synthase
ACO2	1.60	3E − 39	10	Aconitase 2
IDH1	0.62	1E − 70	119	Isocitrate dehydrogenase 1 (NADP +)
IDH2	0.82	3E − 08	47	Isocitrate dehydrogenase 2 (NADP +)
IDH3A	1.52	5E − 13	4	Isocitrate dehydrogenase 3 (NAD +) α
OGDH	1.33	3E − 09	10	Oxoglutarate dehydrogenase
DLD	1.49	1E − 14	8	Dihydrolipoamide dehydrogenase
DLST	1.62	7E − 42	20	Dihydrolipoamide S-succinyltransferase
SUCLG1	1.35	8E − 11	15	Succinate-CoA ligase subunit α
SUCLA2	1.58	2E − 16	4	Succinate-CoA ligase [ADP-forming] subunit β
SUCLG2	1.29	1E − 06	14	Succinate-CoA ligase [GDP-forming] subunit β
SDHA	1.47	8E − 13	4	Succinate dehydrogenase complex Flavoprotein subunit A
SDHB	1.54	7E − 24	24	Succinate dehydrogenase complex iron sulfur subunit B
SDHD	1.37	2E − 07	15	Succinate dehydrogenase cytochrome b small subunit
MDH2	1.34	1E − 21	57	Malate dehydrogenase 2, mitochondrial
Pentose phosphate pathway				
G6PD	1.35	2E − 14	21	Glucose-6-phosphate dehydrogenase
PGLS	0.74	3E − 08	21	6-Phosphogluconolactonase
PGD	1.36	7E − 25	89	Phosphogluconate dehydrogenase
RPE	1.89	7E − 33	13	Ribulose 5-phosphate 3-epimerase
RPIA	1.98	3E − 34	14	Ribose 5-phosphate isomerase A
TKT	1.57	4E − 61	57	Transketolase
TALDO1	1.34	4E − 17	47	Transaldolase 1
Citrate shuttle				
CS	1.18	5E − 07	45	Citrate synthase
ACLY	1.39	4E − 32	119	ATP citrate lyase
MDH2	1.34	1E − 21	57	Malate dehydrogenase 2, mitochondrial
MPC1	2.06	3E − 68	45	Mitochondrial pyruvate carrier 1
PC	0.80	5E − 08	15	Pyruvate carboxylase
Fatty acid synthesis				
ACACA	1.98	3E − 78	7	Acetyl-CoA carboxylase α
FASN	1.77	2E − 91	156	Fatty acid synthase
ACSL1	2.00	2E − 20	2	Long-chain-fatty-acid—CoA ligase 1
ACSL3	1.59	4E − 50	30	Long-chain-fatty-acid—CoA ligase 3
ACSL4	1.92	5E − 18	38	Long-chain-fatty-acid—CoA ligase 4
ACSL5	1.21	1E − 07	28	Long-chain-fatty-acid—CoA ligase 5
ELOVL1	1.33	1E − 09	18	ELOVL fatty acid elongase 1
ELOVL2	0.81	2E − 07	34	ELOVL fatty acid elongase 2
ELOVL5	1.62	3E − 30	30	ELOVL fatty acid elongase 5
HACD2	1.56	2E − 29	15	Very-long-chain 3-hydroxyacyl-CoA dehydratase 2
HACD3	1.28	4E − 13	21	3-Hydroxyacyl-CoA dehydratase 3
Carnitine shuttle				
SLC25A20	0.50	9E − 15	9	Mitochondrial carnitine/acylcarnitine carrier protein
CPT2	0.19	4E − 88	3	Carnitine O-palmitoyltransferase 2, mitochondrial
Fatty acid oxidation				
ACADM	0.51	2E − 36	4	Acyl-CoA dehydrogenase, medium chain
ECHS1	0.81	8E − 10	138	Enoyl-CoA hydratase short chain 1

Sampling of all significantly induced or repressed genes (*p* < 10^−6^; *n* = 3) with robust baseline expression (RPKM > 1) for the following metabolic pathways: glycolysis, citric acid cycle, pentose phosphate pathway, citrate shuttle, fatty acid synthesis, carnitine shuttle, and fatty acid oxidation. A complete list of genes irrespective of their *p* value is provided in Suppl. Tab. 2, including a sampling of all detected NADPH-generating enzymes

Out of nine substantially expressed genes for citrate shuttle components, four genes were significantly upregulated by metformin treatment, among them ATP citrate lyase (ACLY) (1.4-fold), while four genes were unaltered, and one gene was downregulated (Table 6; Suppl. Tab. 2). Fatty acid biosynthesis structural and regulatory genes, in turn, were robustly induced. The mRNAs for acetyl-CoA carboxylase α (ACACA) as well as fatty acid synthase (FASN) were both significantly elevated (2.0-fold and 1.8-fold, respectively), as were the transcripts of most other enzymes in this pathway. Concomitantly, the gene for the breast cancer type 1 susceptibility protein (BRCA1), a potent regulatory inhibitor of ACACA [53], was significantly downregulated following metformin treatment (0.2-fold), whereas the ACACA activator MID1IP1 [60] was upregulated (2.2-fold). These data clearly indicate a coordinated transcriptional effort in metformin-treated HepG2 cells to increase fatty acid biosynthesis.

The majority of genes encoding enzymes of the β-oxidation pathway, especially the more highly expressed genes, were unaltered. Still, two components of short-chain fatty acid metabolism as well as two components of the carnitine shuttle, specifically the mitochondrial carnitine/acylcarnitine carrier SLC25A20 (0.5-fold), were significantly downregulated (Table 6; Suppl. Tab. 2), which could indicate some inhibition of fatty acid oxidation. On the other hand, the gene encoding the regulatory inhibitor of the carnitine shuttle, acetyl-CoA carboxylase β (ACACB) [1], was also significantly downregulated (0.3-fold). In addition, the gene for the autocrine regulatory inducer of hepatic β-oxidation, GDF15 [114], was massively induced (9.7-fold) (Table 3). These contrasting transcriptional changes complicate the prediction of the effective flux through β-oxidation in the HepG2 cell model. Frequently, however, regulatory protein effects tend to prevail over executive enzyme effects in vivo, since the latter are often present in excess and are adaptively tuned by regulatory mediators. This interpretation is supported by in vivo evidence that metformin stimulates hepatic β-oxidation in rodents [104, 105] and counteracts hepatic triglyceride accumulation in non-alcoholic fatty liver disease (NAFLD) patients [89].

Looking at the glycolysis/citric acid cycle/respiratory chain axis, a broad downregulation of glycolysis genes was contrasted by a similarly broad, but milder upregulation of genes for the citric acid cycle, and by a largely unaltered transcription of respiratory chain complex subunits [49]. Compared to the uniform, arguably compensatory induction of all three components, especially glycolysis, in neuronal cells (Table 2), it appears that the response of the HepG2 cells was more selective and differentiated. The downregulation of glycolytic enzymes in these metabolically versatile cells may either reflect a parsimonious reduction to relax the burden on the gluconeogenetic pathway, or an additional regulatory diversion of internalized glucose towards the pentose phosphate pathway to foster NADPH production.

## Discussion

In this study, we describe a coordinated transcriptional induction of genes encoding enzymes that catalyze fatty acid biosynthesis and NADPH production as a response to mitochondrial complex I inhibition. In conjunction with concomitantly facilitated or sustained fatty acid oxidation, a cycle of fatty acid synthesis and degradation emerges that serves as a metabolic mechanism to convert NADPH from the pentose phosphate pathway into FADH_2_, which can ultimately be used to fuel the respiratory chain through the electron-transferring flavoprotein (ETF) complex. The coupling of the pentose phosphate pathway with fatty acid cycling described herein, also referred to as the “NADPH-FADH_2_ axis,” provides an avenue of complete catabolic glucose oxidation that does not produce more NADH than it consumes; hence, it remains neutral with regard to the NADH/NAD^+^ redox pair. In contrast to glycolysis, in which the 24 electrons coming from the oxidation of one molecule of glucose result in the formation of 10 NADH and 2 FADH_2_, glucose oxidation through the NADPH-FADH_2_ axis exclusively produces 12 molecules of FADH_2_ (Table 1; Fig. 3). Since respiratory FADH_2_ oxidation via the ETF complex is independent from complex I activity, the inhibition of this complex is bypassed.

The described pathway of NAD^+^-independent glucose oxidation bypassing complex I is energetically negative, leading to a loss of − 19 ATP per glucose, as opposed to a gain of + 32 ATP per glucose in the coupling of glycolysis, pyruvate dehydrogenase and the citric acid cycle. Hence, the NADPH-FADH_2_ axis is arguably not an evolutionarily selected pathway of household glucose oxidation for bioenergetic purposes, but rather an emergency phenomenon related to glucose detoxification or distress respiration. More specifically, it appears to support mitochondrial respiration despite complex I inhibition in some tissues (e.g., in a compromised liver) at the cost of energetic losses in other tissues whose respiratory chain is still functional (e.g., in adipose tissue). Considering methionine-restricted rats, the reported degree of metabolic inefficiency (approximately 150% food intake per body weight [44, 88]) suggests that about 20% of the ingested glucose are metabolized via the NADPH-FADH_2_ axis, while about 80% remain to be degraded via glycolysis and the citric acid cycle. This estimate fits well to the estimated 15% contribution of the liver to the basal metabolic rate in rats [30]. Notably, all ATP generated by the NADPH-FADH_2_ axis is formed in the liver, whereas all ATP consumed is lost in adipose tissue when the NADPH-FADH_2_ axis is distributed between these two tissues as per the model depicted in Fig. 4. Plausibly, the liver and the gut will be much more frequently affected by complex I inhibition than adipose tissue after ingestion of nutritional toxins that inhibit complex I. Several dozens of highly potent natural complex I inhibitors, mostly from plant and common soil bacterial sources, have been characterized [27, 81], whose occasional ingestion appears inevitable in animal species with a wide food spectrum. In addition to the direct intoxication of complex I by nutritional chemicals, other forms of functional impairment of glycolysis and the NADH-producing axis may also be buffered by the NADPH-FADH_2_ axis, mitigating hepatotoxicity.

Different observations clearly indicate that the described metabolic coupling represents a coordinated physiological response. First of all, the described coupling was transcriptionally induced and was not only passive consequence of metabolite diversion arising from a simple overflow of glucose-6-phosphate into the pentose phosphate pathway [101]. Second, several other pathways of NADPH formation were induced in addition to the pentose phosphate pathway, indicative of a coordinated effort. Third, in the in vivo model, the transcriptional responses were predominantly opposite in liver and adipose tissue, very likely reflecting purposeful organ communication for a common goal (Fig. 4). Superimposition of the induced changes in the liver and in adipose tissue, however, recapitulated the patterns seen in complex I-inhibitor treated cells (Fig. 1; Fig. 3), as further discussed below.

The transcriptional induction of the components of the NADPH-FADH_2_ axis is triggered by an event, namely complex I inhibition, that would be expected to suppress costly pathways like fatty acid synthesis, but rather induce glycolysis and the citric acid cycle to increase ATP production. In fact, such a compensatory response was observed in LUHMES neuronal cells (Table 2), in which MPP upregulated the genes for glycolytic glucose oxidation in addition to the NADPH-FADH_2_ axis. In HepG2 hepatocytes, however, inhibition of complex I with metformin downregulated glycolytic gene transcription (Table 6), as did methionine restriction in the liver in vivo (Table 4; Table 5). We interpret this discrepancy to be due to the relatively undifferentiated phenotype of LUHMES cells, which may allow multiple compensatory responses to occur in parallel. LUHMES cells are a human fetal neuronal cell line that expresses early neuroblast and stem cell markers even after in vitro-differentiation [96] as also adopted in the analyzed experiment [5]. Alternatively, differences might have arisen from the use of unequal complex I inhibitors, namely MPP versus metformin, and from the shorter application of metformin (12 h) compared to MPP (48 h). Irrespectively of the answer to why the three investigated systems showed a heterogeneous response of glycolytic and certain other genes, the striking observation at this point is the common and shared transcriptional upregulation of NADPH production and fatty acid biosynthesis in all systems as the apparent default response to complex I inhibition. Notably, induction of the pentose phosphate pathway has already been described as the primary disease-related metabolic change in genetically complex I-deficient patient fibroblasts [106].

Transcriptional induction of genes encoding the enzymes of a certain metabolic pathway does not necessarily predict metabolic flux. Even if many of the significantly induced enzymes described in this work are generally considered rate-limiting (e.g., G6PD and TKT for the pentose phosphate pathway, and CS, SCL25A1, ACLY and ACACA for fatty acid synthesis), regulatory effects of allosteric inhibitors, enzyme phosphorylation, or substrate and cofactor deficiency may still restrict flux in many cases [24, 52, 82, 85]. Having said this, if a metabolic coupling like the NADPH-FADH_2_ axis was distributed between different tissues or different times of day, many of the potential antagonisms between transcriptional induction and allosteric inhibition would resolve. And indeed, both types of distributional separation seem to be realized in methionine-restricted rats. As shown in Table 4, several rate-limiting enzymes of fatty acid synthesis were reciprocally regulated by methionine restriction in vivo: ATP citrate lyase (ACLY) was significantly downregulated (0.48-fold) in the liver, but significantly upregulated (1.7-fold) in adipose tissue. Moreover, household acetyl-CoA carboxylase (ACACA) was significantly upregulated (3.4-fold) in adipose tissue, whereas regulatory ACACB inhibiting β-oxidation [1] was downregulated (0.46-fold) in the liver. In consequence, several fatty acids and monoacyl glycerides were decreased in the liver, but increased in adipose tissue (Table 5) [87].

Interestingly, another distributional effect has been reported for methionine-restricted rats before, which is the temporal separation of fatty acid synthesis and oxidation. Circadian analysis of respiratory quotients (RQs) has indicated that during the night, the animals performed a net conversion of glucose to fatty acids (denoted by an unusually high RQ > 1), while during daytime, the animals relied almost completely on β-oxidation (denoted by an unusually low RQ ≈ 0.75) despite a largely carbohydrate-based diet (20% glucose, 48% starch and maltodextrin, 8% fat) [44]. These observations are in full agreement with a hepatic or even systemic switch to β-oxidation of fatty acids during the day, which have been freshly synthesized during the night from nutritional glucose in adipose tissue.

In addition to primary complex I inhibition, imposition of β-oxidation in the liver by PPAR agonists [51, 67, 99] may constitute an independent trigger of the NADPH-FADH_2_ axis as described in this study. Ongoing fatty acid catabolism in the liver strongly suppresses glucose oxidation by several allosteric effectors [52], perhaps as efficiently as complex I inhibition, making the liver call for continued fatty acid delivery from adipose tissue via FGF21 and other signals [54, 72]. In response, the adipose tissue will engage in insulin-stimulated de novo lipogenesis as long as sufficient nutritional glucose or lactate are available for this purpose [65, 108]. Therefore, a hepatic switch from glycolysis to β-oxidation can be induced by pull factors (i.e., transcriptionally enforced β-oxidation as per fibrate treatment) and push factors (i.e., curtailed glycolysis due to NADH accumulation after complex I inhibition), indicating a mechanistic convergence of antidiabetic interventions as different as metformin, fibrates, and methionine restriction. Whether the pull side or the push side is more assertive in the induction of the individual changes establishing the NADPH-FADH_2_ axis remains to be determined.

The separation of fatty acid cycling between two different tissues as mentioned before brings the question how the involved metabolites are transported. Transport of albumin-bound fatty acids from adipose tissue to the liver for internalization by fatty acid translocase (CD36) is certainly the default mechanism in the forward direction (Fig. 4). In fact, the genes for CD36 and other pathways of lipid uptake such as VLDL receptors were highly induced in the methionine-restricted liver (Table 4). In the back direction, the acetyl-CoA units generated by hepatic β-oxidation as well as one molecule of NADH per acetyl-CoA unit would have to be returned to the adipose tissue for the formal completion of the cycle. Ketone body production would provide a straightforward answer to the acetyl-CoA issue and to 50% of the NADH to be transported (the synthesis of β-hydroxybutyrate from two acetyl-CoA consumes only one NADH) (Fig. 4). As it happens, adipose tissue has long been known to utilize ketones for the synthesis of fatty acids, and this utilization is stimulated by glucose and insulin [97], i.e., in the carbohydrate-fed state as realized in the methionine-restricted rats. Experimentally, a significant increase in β-hydroxybutyrate after methionine restriction has been evidenced for serum (2.4-fold) and skeletal muscle (3.2-fold) in rats (Table 5) [87], and for serum in genetically obese mice 76. Regarding the remainder of the NADH, transport as lactate (generated from pyruvate under conditions of a high NADH/NAD^+^ ratio) is a potential option since lactate is a preferred substrate for lipogenesis in adipose tissue, whose utilization is again stimulated by glucose, i.e., in the fed state [58, 65]. Experimentally, lactate levels have not been found to be persistently elevated in the methionine restriction model in vivo. However, the significant induction of lactate dehydrogenase mRNA in adipose tissue (1.7-fold) and skeletal muscle (2.6-fold) of the methionine-restricted rats (Table 4) may indicate a chronically increased utilization of circulating lactate by these tissues. Consistently, the reduction of pyruvate in the liver (0.43-fold) and in serum (0.24-fold), resulting in significantly elevated lactate/pyruvate (L/P) ratios of 1.6 and 3.7, respectively (Table 5), may reflect a rapid, NADH-driven conversion of pyruvate to lactate for export from the liver to lactate-consuming tissues. The L/P ratio has been used as clinical biomarker of hereditary complex I deficiency before [69]. After all, both lactate and ketone body production by the liver have long been known to be induced by metformin and other biguanides in animals and man [71, 104, 105].

The endocrine coordination of the NADPH-FADH_2_ axis among different tissues is likely driven by mitochondrial stress-induced cytokines (mitokines) including FGF21 50 and GDF15 [15]. As summarized in Table 3, FGF21 and GDF15 were widely induced in the investigated models. FGF21 is an endocrine signal that strongly stimulates glucose uptake in adipocytes [59]. At the same time, it triggers the provision of free fatty acids by adipose tissue preferably in the fed state, i.e., under non-fasting conditions 50. This ostensibly paradoxical behavior becomes comprehensible in a scenario in which hepatic complex I is temporarily inhibited by a food-borne toxin. To forestall irreversible hepatic damage due to ATP depletion, the NADPH-FADH_2_ axis is activated, by which the liver is supplied with life-saving fatty acids, while adipose tissue utilizes and degrades the excess glucose. Since FGF21 is a target gene of PPARα [54, 72], it may plausibly represent a downstream mediator of fatty acid cycling as induced by fibrates [82] and other triggers of PPARα activation, among them methionine restriction [86] and metformin treatment [3, 73]. Importantly, FGF21 knock-out mice have been shown to lack several key aspects in their response to methionine restriction compared to wild-type mice [34, 107,31], especially in regard to energy expenditure and insulin sensitivity. In the liver, though, gene expression modulation by methionine restriction was unaltered in FGF21 knock-out mice, suggesting additional regulators of the NADPH-FADH_2_ axis in this organ. We propose that GDF15 may be critically involved, even if methionine restriction has not been investigated in GDF15 knock-out mice yet.

GDF15 is a general indicator of mitochondrial metabolic insufficiency and substrate demand [15] and was induced about tenfold within 12 h of moderate-dose metformin treatment in HepG2 cells (Table 3). Furthermore, GDF15 has been shown to promote fatty acid β-oxidation and ketogenesis in the liver in response to bioenergetic deficits [114]. At the same time, GDF15 directly signals to the brain to avoid food intake [84] and stimulates vomiting and nausea [13]. The paradoxical avoidance of food intake and the induction of emesis despite a bioenergetic deficit may seem expedient after nutritional intoxication with one of the many mitochondrial toxins present in the environment [27]. Potentially, GDF15 monitors mitochondrial integrity in particularly exposed tissues (liver), sensitive tissues (placenta), or sensitive states (infancy) [15], and if necessary, orchestrates a reversible switch from external, contaminated energy sources (food) to internal, safe energy sources (stored triacylglycerols).

## Conclusion

Hepatic complex I inhibition leads to a shutdown of glycolysis and the citric acid cycle by NADH, for which the liver switches to β-oxidation as main energy source. Therefore, the liver sends a signal to the adipose tissue to be reliably provided with fatty acids, whereupon the adipose tissue initiates the synthesis of fatty acids from nutritional glucose before resorting to stored triacylglycerols. This physiological adaptation is induced in adipose tissue by FGF21. Hence, hepatic fat hunger signaling turns adipose tissue into a highly glucose-metabolizing organ, which accounts for the diabetes-resistant phenotype that ensues.

### Supplementary Information

Below is the link to the electronic supplementary material.

Supplementary file1 (XLSX 17 KB)

Supplementary file2 (XLSX 17 KB)

## Data Availability

All raw data analyzed in this study were adopted from [5], [49] and [87]. Processed data are available from the corresponding author upon request.
